# Catchment area rates of involuntary care and subsequent patient morbidity and mortality in Norway

**DOI:** 10.1192/j.eurpsy.2024.229

**Published:** 2024-08-27

**Authors:** O. Nyttingnes

**Affiliations:** ^1^Health Services Research Unit, Akershus University Hospital, Lørenskog; ^2^Centre for Research and Education in Forensic Psychiatry, Haukeland University Hospital, Bergen, Norway

## Abstract

**Introduction:**

Mental health legislation allows for involuntary care of patients with severe mental disorders, assuming it improves health and reduces risk. Professionals have warned against potentially adverse effects of recent initiatives to heighten involuntary care threshold, such as CRPD and national coercion-reduction strategies. We have not found that the impact of high thresholds for involuntary care have been studied.

**Objectives:**

Our aim was to use national data from Norway to test implications of the hypothesis that areas with lower levels of involuntary care show higher levels of morbidity and mortality in their severe mental disorder populations compared to areas with higher levels. We pre-specified five models of how such adverse effects could manifest in national register data.

**Methods:**

Using national register data, we calculated standardized (by age, sex, and urbanicity) involuntary care ratios across Community Mental Health Center areas in Norway. For patients diagnosed with severe mental disorders (ICD10 F20-31), we tested whether lower area ratios in 2015 interacted with 1) case fatality over four years, 2) an increase in inpatient days, and 3) time to first episode of involuntary care over the following two years. We also assessed 4) whether area ratios in 2015 predicted an increase in the number of patients diagnosed with F20-31 in the subsequent two years and whether 5) standardized involuntary care area ratios in 2014–2017 predicted an increase in the standardized suicide ratios in 2014–2018.

**Results:**

We included 21481 patients with either an F20-31 diagnosis, an episode of involuntary care in 2015, or both. The standardization variables age, sex, and urbanicity explained 70.5% of the variance in raw rates of involuntary care, and the remaining extremal quotient was 2.5. Age and sex predicted case-fatality, but involuntary care-rate was insignificant. Patients with F20-31 and no involuntary care episode in 2015 showed a steady reduction in inpatient days the following years, but not significantly related to the area’s involuntary care rates. For the same sample, these rates did not predict the time to an episode of involuntary care. The area’s involuntary care rate in 2015 did not predict *changes* in the number of patients in treatment for a diagnosis of F20-31 from 2015-2017. Finally, the area’s involuntary care rate from 2014-2018 explained 1.2% of the variance in suicides in 2014-2019 in the area.

**Conclusions:**

In the models, we found no significant associations between low standardized catchment area rates of involuntary care and the pre-specified outcomes. This raises questions about some assumptions in mental health legislation and merits further research.

**Disclosure of Interest:**

None Declared

